# Work-life interface and intention to stay in the midwifery profession among pre- and post-clinical placement students in Canada

**DOI:** 10.1186/s12960-020-00509-4

**Published:** 2020-09-22

**Authors:** Farimah HakemZadeh, Elena Neiterman, James Chowhan, Jennifer Plenderleith, Johanna Geraci, Isik Zeytinoglu, Derek Lobb

**Affiliations:** 1 School of Human Resource Management, York University, 4700 Keele St, Toronto, ON M3J 1P3 Canada; 2 School of Public Health and Health Systems, University of Waterloo, 200 University Ave W, Waterloo, ON N2L 3G1 Canada; 3 DeGroote School of Business, McMaster University, 1280 Main St W, Hamilton, ON L8S 4L8 Canada; 4 College of Midwives of Ontario, 21 St Clair Ave E #303, Toronto, ON M4T 1L9 Canada; 5 Department of Obstetrics and Gynecology, McMaster University, 1280 Main St W, Hamilton, ON L8S 4L8 Canada

**Keywords:** Midwifery, Intention to stay in the profession, Work/life conflict, Work/life enhancement, Work/life balance

## Abstract

**Background:**

Midwifery students’ intention to stay in the profession can be influenced by how the interface of their work and personal life is affected by their clinical placement experience. The purpose of this study is to compare the intention to stay in the midwifery profession and its association with three work/personal life interface constructs among pre- and post-clinical placement midwifery students in Canada. The constructs investigated are work interference with personal life, personal life interference with work, and work/personal life enhancement.

**Methods:**

Quantitative cross-sectional data were collected through two separate online surveys completed by pre- and post-clinical placement students. In total, 456 midwifery students attending six different midwifery education programs responded to the surveys.

**Results:**

Compared to pre-clinical placement students, post-clinical placement students had significantly lower intention to stay in the profession. For pre-clinical placement students, higher personal life interference with work was significantly associated with lower intention to stay in the profession. For post-clinical placement students, higher work interference with personal life was associated with lower intention to stay in the profession. We did not find any significant relationships between work/personal life enhancement and intention to stay in the profession in pre- or post-clinical placement students.

**Conclusion:**

Pre- and post-clinical placement students have different intentions to stay in the profession. For pre-clinical placement students, those who report that their personal lives highly interfere with work are less likely to want to stay in the midwifery profession. Post-clinical placement students reported that when working interfered with their personal lives they were less likely to want to stay in the profession. Our findings highlight the importance of offering students a realistic preview of the required commitment, workload, and personal involvement in the midwifery profession prior to applying or accepting a spot in a midwifery education program. Furthermore, facilitating the development of skills to better manage the expectations in midwifery work and personal lives might help with maintaining positive intentions to stay in the profession.

## Background

Midwifery, a relatively newly regulated profession in Canada, adopts a model of care in which midwives are autonomous primary care providers, offering continuity of care to their clients during pregnancy, birth, and up to six weeks in the postpartum period [1]. While countries with comparable midwifery practices, such as the Netherlands and New Zealand, have more than 21 (data from 2013) and 49 (data from 2017) professionally active midwives per 1000 live-births, respectively, Canada has less than 5 (data from 2017) [2]. As a result, many provinces and territories in Canada cannot fulfil the maternity care needs of their populations. Countries such as the United Kingdom [3, 4] and Australia [5] are also warning of the severe shortage of midwives. According to the World Health Organization, a shortage of nine million midwives and nurses is anticipated globally through to 2030 [6]. Understanding this workforce shortage and designing potential interventions also require exploring student midwives’ intention to stay in the profession throughout their training.

The path towards becoming a midwife in Canada starts with specialized professional training in universities. Compared to the retention rate of university students in general, and students pursuing a career in health professions in particular, midwifery students in Canada have a lower retention rate [7]. Retention in midwifery education is also a concern in other countries such as Australia [8] and the United Kingdom [9]. Ignoring this problem would negatively affect the supply of midwives and effective health human resource planning. This paper focuses on the work/personal life interface and intention to stay in the profession by examining the perspectives of pre- and post-clinical placement midwifery students in Canada. This is particularly motivated by the argument that when students apply to study midwifery, they do not know what being a midwife actually involves until after they enter the clinical placement portion of their studies [10].

### Students’ intention to stay and clinical placement

For this research, the concept of intention to stay in the profession is defined as an individual’s conscious willingness to remain in a certain profession [11]. This study of the intention to stay is informed by the theory of planned behaviour [12], which assumes that behavioural intention is one of the most immediate predictors of the behaviour. This approach has theoretical and empirical support, suggesting that low intention to stay is a critical mediating variable in the decision process that results in the decision to leave [13, 14].

Currently, there are six active midwifery education programmes in Canada. All six programmes (Table 1) offer students in-class courses and several clinical placements to facilitate the development of required skills.

**Table 1:** List of active midwifery education programmes in Canada

School	Degree	Language	Number of accepted students per year
Mount Royal University	Bachelor of Midwifery	English	12
University of British Columbia	Bachelor of Midwifery	English	20
Laurentian University	Bachelor of Health Sciences	English and French	30
McMaster University	Bachelor of Health Sciences	English	30
Ryerson University	Bachelor of Health Sciences	English	30
Université du Québec à Trois-Rivières	Bachelor of Science	French	24

During clinical placements, students engage in the daily activities of a practising midwife but are being supervised. These activities include “conducting prenatal visits, taking medical histories, and conducting physical exams of clients and their babies” [15]. Midwifery education programmes emphasize that during placements, students will work alongside a preceptor so that “when the midwife is in the clinic, the student is in the clinic. When the midwife’s pager goes off at 2 a.m. for a woman in labour, the student’s does too. When the midwife is driving out under the stars, or perhaps the northern lights to attend a birth at 4 a.m., the student is also on her way to the birth” [16]. Research has demonstrated that students obtain a preview of their future work environments through these clinical placements [17, 18]. These realistic previews provide students with information about both the positive and negative aspects of work and life as a practising midwife and allow them to make more informed decisions about their careers [19].

For both midwifery and nursing students, clinical placement has been identified as a “tipping point” [20] in students’ intentions to stay or leave the programme [10, 21–23]. Empirical evidence confirms that clinical placement affects career decisions [24] including midwifery as a career choice [23] and intention to stay [22, 25, 26].

### Negative and positive work/personal life interface

This study is also informed by role theory [27], which suggests that to understand the work attitudes of individuals, such as their intention to stay in a profession, consideration must be given to the differing roles in an individual’s life, both at work and outside of work. Midwifery is a demanding profession with high expectations of professional performance and levels of responsibility and often requires long, irregular working hours [28–30]. Given that the vast majority of midwives are women, these expectations need to be balanced with traditional gender roles related to familial responsibilities. Therefore, the work-personal life interface is one of the key factors that might influence students’ intentions to stay in the profession [31–33], particularly after they are exposed to the realities of the midwifery profession through their clinical placement. The work/personal life interface has both positive and negative sides that can impact intention to stay in the profession differently.

The negative side of work/personal life interface is informed by the scarcity hypothesis [34] and strain theory [35]. The scarcity hypothesis argues that because an individual’s time and physical and mental capacity are not unlimited, she makes trade-off decisions about how to allocate these resources across her different roles [34]. When demands of these resources among different roles become incompatible, they will put strain and pressure on the individual [35]. Strain theory, complemented by the demands-resources model, has been applied to understanding the work/personal life interface [36] by arguing that the incompatibility of demands and resources can be studied in two directions: work demands from the resources allocated to one’s personal life (work interference with personal life (WIPL)) and personal life demands from the resources allocated to one’s work (personal life interference with work (PLIW)).

In addition to organizational and life-related outcomes of the work/personal life interface, career-related outcomes have been of interest to researchers. For example, Peluchette [37] argues that those experiencing higher levels of work/personal life conflict report lower subjective career success. Students in midwifery programs are being trained for a midwifery career, and thus, experiencing work/personal life interference might affect their perception of future career success and intention to stay in the profession.

Despite its work/personal life interface challenges, midwifery is suggested to be a meaningful profession that provides the opportunity to experience a fulfilling career helping women bring life into the world [38]. Student midwives can also potentially experience the positive side of work/personal life interface. For example, a recent review on the reasons that practising midwives stay in the profession [39], while pointing out that the literature on the topic is scarce, suggests that midwives stay in their professions because they “feel proud and privileged to be a midwife, and protect normality in pregnancy and birth” and that their “passion for midwifery” helps them to tolerate the difficulties. Therefore, it is essential to capture the positive work/personal life interface and its association with intention to stay in the profession in the context of midwifery education. We argue that work or personal life can have enhancing effects on one another, making it easier to participate in one and manage the other. This positive perception can influence individuals’ intentions to stay in their professions.

Allen [40] identifies expansion theory [41] as the basis of conceptualizing the positive interface of work and personal life. This theory suggests that, through assuming different roles, an individual might expand time resources, along with their physical and mental capacity. Furthermore, there can be a spillover of happiness and success between work and other domains [42]. Concepts such as positive spillover [43], work/personal life facilitation [44] (where skills learned in one domain foster performance in another), and work/personal life enrichment [45] (where the quality of life is improved due to the transfer of resources from one role to the other) are often used to study this phenomenon. We use the term work/personal life enhancement (WPLE) [40] to generally refer to the positive benefits of work and personal life roles on one another. WPLE was shown to be positively related to family and job satisfaction, physical and mental health, and affective organizational commitment [46].

Previous studies have reflected on encountering reality shock and doubting career choices during initial clinical experiences in similar professions to midwifery, such as nursing [47]. Similarly, we argue that differences in perceptions of the interface of work and personal life in midwifery students at different stages in their professional training are associated with their intention to stay in the profession and this association is moderated by midwifery students’ clinical placement experience. The presented theories and literature review then enable us to make the following hypothesis:

*The negative effects of work interference with personal life and personal life interference with work, and the positive effect of work/personal life enhancement, on intention to stay in the midwifery profession are negatively moderated by students’ participation in clinical placement.*

## Methods

### Study design, recruitment, and enrolment of study participants

This study is based on a cross-sectional design in two times. From 2016 to 2019, two surveys were distributed online to first year and second year midwifery students across Canada: (1) pre-clinical placement survey and (2) post-clinical placement survey, which captured the perspectives of two different cohorts of students at pre- and post-clinical placement stages of their education throughout the years of data collection. Both surveys included questions on intention to stay in the midwifery programme, work interference with personal life, personal life interference with work, work/personal life enhancement, and some demographic questions. Details on the number of questions for each construct, their wording, response options, and their reliability are provided in the measurement section of this paper. The study and its data collection approach, including all questions, were reviewed and approved by the research ethics boards of universities offering midwifery education programmes and at the co-authors’ affiliated universities. Students who participated in the study were entered into an annual draw for one of three $50 CAD e-gift cards.

Each year, midwifery programmes across Canada admit 146 students. Since the data were collected over a period of 3 years, our target population was a maximum of (3 × 146 =) 438 students for each pre- and post-clinical placement surveys.

### Sample

Overall, we received 155 and 213 unique responses for our pre- and post-clinical surveys, respectively. Additionally, 88 students responded to both surveys. This is because study invitations and reminders were sent out to students at different times in their programmes as schools across Canada schedule their placements at different times of the year. In order to keep the surveys’ responses unique and avoid possible bias by deleting all these 88 responses, a decision was made to keep one of their responses (either pre- or post-clinical) and to make our two cohort groups equal for statistical analysis. At random, we included the pre-clinical placement survey responses from 73 of these 88 students and post-clinical placement survey responses from 15 of them. Thus, the final sample used in the analysis has *n* = 456 responses, with equal numbers (*n* = 228) in both pre- and post-clinical placement surveys (i.e. for the pre 155 + 73 = 228 and for the post 213 + 15 = 228, respectively). This gives us a conservative response rate of (228/438=) 52% for each pre- and post-clinical placement surveys.

Participants were asked to answer demographic questions about their age, whether or not they had children, their marital status, prior education, and prior career. We sought this data because age [48, 49], prior education and career [49, 50], marital status , and children can influence one’s perception of work/personal life interface and its association with attitudes and intentions.

### Measures

The intention to stay in the profession scale was adapted from Lyons’ [51] three-item scale and slightly modified in consultation with the research advisory board of the project. Participants were asked to select their level of agreement with each of the following items on a 5-point Likert scale from 1 = strongly disagree to 5 = strongly agree: (1) If I were completely free to choose, I would prefer to work as a midwife; (2) I would like to stay in the midwifery programme until completed; and (3) If I had to leave the midwifery program for a while (for example because of personal/family reasons), I would return to it. Items were then summed to create a scale (Cronbach’s alpha = 0.73).

Fisher’s [52] scales were incorporated to capture work/personal life interface (work interference with personal life (WIPL), personal life interference with work (PLIW), and work/personal life enhancement (WPLE)). Some of the items included in the survey were as follows: “My personal life suffers because of studying as a midwife” (WIPL); “My personal life drains me of energy for studying” (PLIW); “My personal life gives me energy for my studies” (WPLE); and “My studies give me energy to pursue personal activities” (WPLE). Participants were asked to select their level of agreement with each statement based on a Likert scale from 1 = strongly disagree to 5 = strongly agree. Work interference with personal life, personal life interference with work, and work/personal life enhancement showed the following Cronbach’s alphas respectively, 0.93, 0.80, and 0.69. The descriptive statistics for these key scales and the correlations between all variables are presented in Table 2.

**Table 2:** Correlations between variables and intention to stay

	Mean	St. Dev.	1	2	3	4	5	6	7	8	9	10
1. Intention to stay	4.54	0.61	*0.73*									
2. Work interference with personal life	3.77	0.87	− 0.32***	*0.93*								
3. Personal life interference with work	2.40	0.82	− 0.14***	0.27***	*0.80*							
4. Work-personal life enhancement	3.04	0.69	0.18***	− 0.51***	− 0.30***	*0.69*						
5. Age (29 plus)^a^	0.46	0.50	− 0.12**	0.24***	0.14***	− 0.03						
6. Prior education (undergrad or greater)^a^	0.59	0.49	− 0.13***	− 0.09**	− 0.08*	0.14***	0.18***					
7. Previous career^a^	0.49	0.50	− 0.04	0.23***	0.07	0.00	0.55***	0.14***				
8. Has children^a^	0.36	0.48	− 0.07	0.19***	0.26***	− 0.01	0.59***	0.05	0.44***			
9. Married or living with a partner, or common-law^a^	0.53	0.50	− 0.10**	0.12***	0.03	0.06	0.42***	0.16***	0.34***	0.43***		
10. Post-clinical placement^a^	0.50	0.50	− 0.21***	0.35***	− 0.04	− 0.15***	0.10**	0.01	0.01	0.03	0.01	

Cronbach’s alphas are presented in italic font. *n* = 456

*p < .10

**p < .05

***p < .01

^a^These variables are binary variables, and as such, their means indicate the percentage of participants with a value equal to 1, where the reference categories of zero are presented in detail in Table 3. For the age variable, we coded 18 to 28 as 0 and 29+ as 1. For prior education, we assumed that having the choice of another career due to having a previous degree might have an impact on how intention to stay in the profession may vary between individuals. Therefore, a completed undergraduate or graduate degree was coded as 1 and all others were coded as 0

### Analysis

Three standardized regression models were estimated using a hierarchical approach to enable model comparisons and more effective testing of our hypothesis. First, in model 1, we tested whether our control variables, such as age (29+), having another university degree, previous career, having children, and marital status (being married, living with a partner, or in a common-law relationship), were associated with intention to stay in the profession. In addition to the variables in model 1, model 2 looked at the association of work interference with personal life, personal life interference with work, and work/personal life enhancement with intention to stay in the profession. Model 3 directly tested our hypothesis about the moderating effect of clinical placement on the relationship between work/personal life interface variables (WIPL, PLIW, WPLE) and intention to stay in the profession. To facilitate an enhanced interpretation of the total effects of the three work/personal life interface variables in pre- and post-clinical placement cohorts, margin analyses were performed enabling predictions of the fitted model at fixed values to be estimated. All analyses were completed using Stata 14.

## Results

In our pre-clinical placement survey, the average age was 29, with 53.9% younger than 28 years old. Furthermore, 58.8% had either an undergraduate or graduate degree and 48.7% had a prior career before starting midwifery education. Moreover, 53.6% of them were married, living with a partner, or in a common-law relationship. In our post-clinical placement survey, the average age was 31, with 49.1% younger than 28 years old. Also, 60.1% at least had an undergraduate degree and 50.0% had another career before starting midwifery education. Finally, 53.9 % were married, living with a partner, or in a common-law relationship. Further details are provided in Table 3.

**Table 3:** Demographic characteristics of participants and coding of binary variables

Demographic characteristic	Full sample (*n* = 456) % (95%CI)	Pre-clinical placement (*n* = 228) % (95%CI)	Post-clinical placement (*n* = 228) % (95%CI)
Age			
18–22	11.4 (8.5–14.3)	14.5 (9.9–19.1)	8.3 (4.7–11.9)
23–28	42.5 (38–47.1)	44.3 (37.8–50.8)	40.8 (34.4–47.2)
29–34	22.1 (18.3–26)	21.5 (16.1–26.9)	22.8 (17.3–28.3)
35–44	19.3 (15.7–22.9)	16.7 (11.8–21.5)	21.9 (16.5–27.3)
45+	4.6 (2.7–6.5)	3.1 (0.8–5.3)	6.1 (3–9.3)
Prior education			
None	6.1 (3.9–8.4)	5.7 (2.7–8.7)	6.6 (3.3–9.8)
Some undergraduate courses	21.1 (17.3–24.8)	22.4 (16.9–27.8)	19.7 (14.5–24.9)
College	13.4 (10.2–16.5)	13.2 (8.7–17.6)	13.6 (9.1–18.1)
Undergraduate degree	46.9 (42.3–51.5)	46.1 (39.5–52.6)	47.8 (41.3–54.3)
Graduate education	12.5 (9.5–15.5)	12.7 (8.4–17.1)	12.3 (8–16.6)
Previous career			
No	50.9		
Yes	49.1 (44.5–53.7)	48.7 (42.1–55.2)	49.6 (43–56.1)
Has children			
No	64.0		
Yes	36 (31.5–40.4)	34.6 (28.4–40.9)	37.3 (31–43.6)
Marital status			
Married or living with a partner/common-law	53.3 (48.7–57.9)	52.6 (46.1–59.2)	53.9 (47.4–60.5)
Single	34.6 (30.3–39)	35.1 (28.8–41.3)	34.2 (28–40.4)
Divorced/separated/widowed	4.2 (2.3–6)	4.8 (2–7.6)	3.5 (1.1–5.9)
Other	7.9 (5.4–10.4)	7.5 (4–10.9)	8.3 (4.7–11.9)

Model 1 produced an adjusted *R*^2^ = 0.06, *F*(6,449) = 5.55, *p* < 0.01; see Table 4. Among the demographic variables, only education showed a significant negative relationship with intention to stay (*B* = − 0.23, *p* < 0.05). This model showed that post-clinical placement students had a lower intention to stay in the profession (*B* = − 0.40, *p* < 0.01) compared to the pre-clinical students.

**Table 4:** Standardized regression coefficient models (ordinary least squares technique)

	Model 1		Model 2		Model 3	
	Coef.	Std. Err.	Coef.	Std. Err.	Coef.	Std. Err.
Dependent variable						
Intention to stay						
Independent variables						
Work interference with personal life (WIPL)			− 0.27***	0.06	− 0.06	0.08
Personal life interference with work (PLIW)			− 0.08*	0.05	− 0.15**	0.07
Work/personal life enhancement (WPLE)			− 0.03	0.05	0.08	0.08
WIPL × post-clinical placement					− 0.42***	0.11
PLIW × post-clinical placement					0.13	0.10
WPLE × post-clinical placement					− 0.09	0.11
Control variables						
Age (29 plus)	− 0.14	0.13	− 0.07	0.12	− 0.29	0.18
Education (undergrad or greater)	− 0.23**	0.95	− 0.33***	0.09	− 0.10	0.13
Previous career	0.08	0.11	0.16	0.11	0.14	0.15
Has children	− 0.01	0.12	0.07	0.12	0.17	0.18
Marital status (being married, living with a partner, or in a common law relationship)	− 0.12	0.10	− 0.13	0.10	− 0.04	0.14
Post-clinical placement	− 0.40***	0.09	− 0.21**	0.09	− 0.03	0.17
Age × post-clinical placement					0.50**	0.25
Education × post-clinical placement					− 0.40**	0.18
Previous career × post-clinical placement					− 0.08	0.22
Children × post-clinical placement					− 0.14	0.24
Marital status × post-clinical placement					− 0.17	0.20
Constant	0.43***	0.10	0.30***	0.10	0.25**	0.12
*F*	5.55***		8.97***		6.19	
Adjusted *R*-square	0.06		0.14		0.16	

*n* = 456

*p < .1

**p < .05

***p < .01

Model 2 produced an adjusted *R*^2^ = 0.14, *F*(9,446) = 8.97. Both work interference with personal life and personal life interference with work had significant negative relationships with intention to stay in the profession (WIPL *B* = − 0.27, *p* < 0.01; PLIW *B* = − .08, *p* < 0.10). However, our model did not show a significant relationship between work/personal life enhancement and intention to stay in the profession.

Model 3 showed an adjusted *R*^2^ = 0.16, *F*(17,438) = 6.19, *p* < 0.01). For pre-clinical placement students, there was a significant relationship between personal life interference with work and intention to stay in the profession (*B* = − 0.15, *p* < 0.05), while for post-clinical placement students, there was a significant relationship between work interference with personal life and intention to stay in the profession (*B* = − 0.42, *p* < 0.01).

**Fig. 1:**
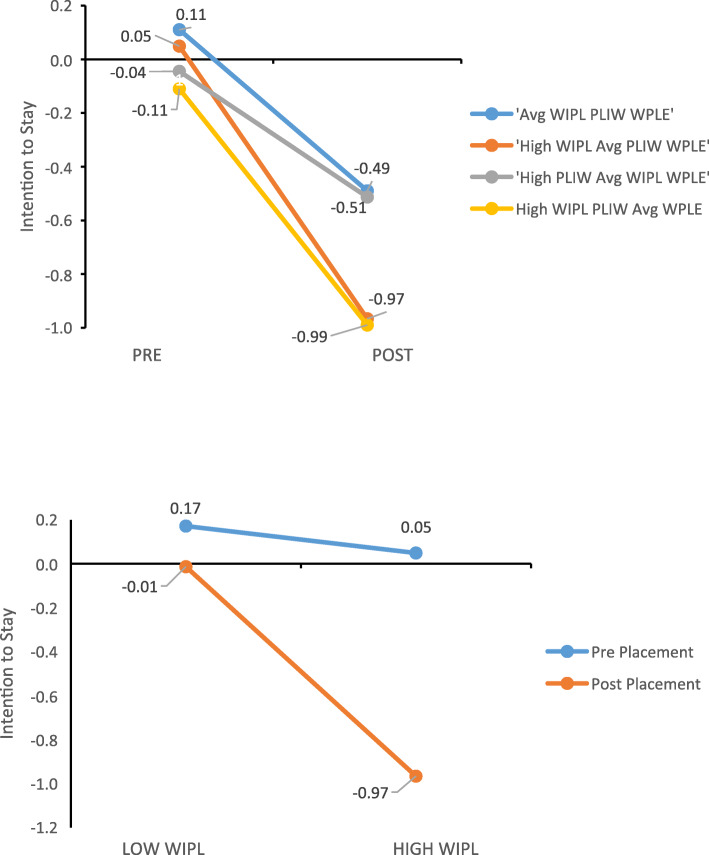
Margin analysis graphs

We kept age, education, and other control variables in the analysis of the margins (with the base case being age less than 29 years, undergraduate degree, no prior career, no children, and married) and calculated adjusted predictions of intention to stay. Results are presented in graphs 1 and 2 (Fig. 1). The intention to stay in the profession outcomes are substantially lower for post-clinical placement compared to pre-clinical placement students, and this is the case when the three work/personal life interface variables are considered at their average values and for other sensitivity explorations included in the figure. These differences are particularly significant for students who are experiencing high work interference with personal life while experiencing average personal life interference with work and work personal life enhancement or experiencing high work interference with personal life and high personal life interference with work while experiencing average work/personal life enhancement. For post-clinical placement students, there is a significantly lower outcome in the association between work interference with personal life and intention to stay in the profession.

## Discussion

The findings of this study suggest that compared to pre-clinical placement midwifery students, post-clinical placement students have a significantly lower intention to stay in the profession. Our data also reveal that in both pre- and post-clinical placement cohorts, midwifery students’ perception of the interface of work and personal life is associated with their intention to stay in the profession. However, the nature of this association is different between the two cohorts. For pre-clinical placement students, personal life interference with work seems to matter, while for post-clinical placement students, it is the interference of work with personal life that significantly influences intention to stay in the profession.

### Implications for policy, practice, and research

The differences in intention to stay in the profession between pre- and post-clinical placement students and the effects of work/personal life interface constructs on intention to stay highlight the importance of regular monitoring of these constructs among students. In terms of health human resource planning, understanding the differences in intention to stay in the profession between pre- and post-clinical placement students can provide policymakers with a better estimate of the number of midwifery students who intend to graduate and become registered practising midwives. Furthermore, as work/personal life interface was shown to be associated with intention to stay in the midwifery profession for midwifery students, health human resources policymakers can develop strategies to support students to maintain a healthy work/personal life interface as midwives.

As this study highlights the lower intention to stay in the profession in post-clinical placement students compared to pre-clinical placement students, we suggest midwifery education programmes consider designing a realistic job preview component in their student recruitment. A realistic preview of the midwifery profession can help students to self-select and have realistic expectations about the midwifery profession. Furthermore, educators can add time-management skills to their programmes’ learning objectives to help students effectively manage the interface of their work and personal lives.

### Recommendations

In a profession, such as midwifery, where the supply of skilled and competent practitioners is critical for providing effective and equitable healthcare services to the population, intention to stay in the profession and factors associated with it need to be continuously monitored. The insights obtained during this study can guide both health human resource planning as well as decisions regarding the type and timing of the required interventions to maintain a high intention to stay in the profession. In this study, we focused on the interface of work and personal life as a factor associated with pre- and post-clinical placement students’ intention to stay in the profession. Preceptors and midwives who mentor and train students during their clinical placements are invaluable allies to ensure that students effectively learn how to manage the interface of their work and personal life as a midwife.

This study has a few limitations. First, while directionality of the negative spill-over effect between work and personal life was explored through two distinct constructs of work interference with personal life and personal life interference with work, the work/personal life enhancement scale did not separately capture work enhancement of personal life and personal life enhancement of work. Second, as the data were cross-sectional, we cannot make causal inferences regarding the effects of clinical placement on student midwives’ intention to stay in the profession. Therefore, we have only been able to explore cohort effects which hint at causality. Third, while the possibility of social desirability bias was reduced through anonymous survey administration, the concern for positive feedback bias remains. Student midwives might consider negative answers to the intention to stay in the profession items as a threat to their self-image and identity as a future midwife.

It is worth noting that midwifery programmes across the world structure the clinical placement of their midwifery education programmes differently. For example, some programmes structure their clinical placements in one or several full-time blocks (e.g. for the duration of 36 weeks) while other programmes might have several weekdays dedicated to in-class courses and others to clinical placement [53]. Therefore, the scheduling of the clinical placement component of midwifery education programmes in Canada should be taken into consideration in interpreting and applying the findings of this study.

Future research with longitudinal data on the changes in career intentions of midwives and other health professionals throughout their career trajectory can better clarify the causal effects of events such as clinical placement on their intention to stay in their professions. Moreover, investigations into characteristics of clinical placements that can positively or negatively affect perceptions of work/personal life interface constructs or intention to stay in the profession can further develop our theoretical and practical insights into this issue.

## Conclusion

The analysis of our data from 456 midwifery students in Canada shows that post-clinical placement students have significantly lower intention to stay in the profession compared to pre-clinical placement students. Furthermore, for pre-clinical placement students, higher interference of personal life with work is significantly associated with lower intention to stay. For post-clinical placement students, it is the higher interference of work with personal life that is associated with lower intention to stay in the profession. Our data further reveal that among post-clinical placement students, those who have completed another degree before entering the midwifery education programme have significantly lower intention to stay in the profession. While age is negatively associated with intention to stay in pre-clinical placement students (although not statistically significant), it is positively associated with intention to stay in post-clinical students.

## Data Availability

The dataset this paper uses is the original data collected and owned by Drs. Zeytinoglu, Hakem-Zadeh, Neiterman, and Lobb. This data can be available only after the owners have completed using the data for their submissions (journal articles and other media outputs). The statistical output that this paper is based on can be available for review, from the corresponding author, upon request.

## Abbreviations

WIPL: Work interference with personal life
PLIW: Personal life interference with work
WPLE: Work/personal life enhancement

## References

[1] Malott AM, Davis BM, McDonald H, Hutton E (2009). Midwifery care in eight industrialized countries: how does Canadian midwifery compare?. J Obstet Gynaecol Can.

[2] Organization for Economic Co-operation (OECD. Stat) (2019). Health care resources: midwives.

[3] England short of almost 2,500 midwives, new birth figures confirm. RCM. [Cited 2020 Apr 13]. Available from: https://www.rcm.org.uk/news-views/rcm-opinion/2019/england-short-of-almost-2-500-midwives-new-birth-figures-confirm/.

[4] Wooller BS (2018). Nearly one in four women in labour left alone due to shortage of doctors and midwives, report reveals. The sun.

[5] Calderwood K, Miskelly G (2018). Too many patients, not enough care: NSW’s looming nursing crisis. ABC news.

[6] World Health Organization. Nursing and midwifery. [Cited 2020 Apr 29]. Available from: https://www.who.int/news-room/fact-sheets/detail/nursing-and-midwifery.

[7] Wilson R, Eva K, Lobb DK (2013). Student attrition in the Ontario midwifery education programme. Midwifery..

[8] Carolan MC, Kruger GB (2011). Concerns among first year midwifery students: towards addressing attrition rates. Contemp Nurse.

[9] Astrup J. Blown off course. In: Royal College of Midwives Magazine. [Cited 2020 Apr 21]. Available from: https://www.rcm.org.uk/news-views/rcm-opinion/blown-off-course/.

[10] Green S, Baird K (2009). An exploratory, comparative study investigating attrition and retention of student midwives. Midwifery..

[11] Tett RP, Meyer JP (1993). Job satisfaction, organizational commitment, turnover intention, and turnover: path analyses based on meta-analytic findings. Pers Psychol.

[12] Fishbein M, Ajzen I (1974). Attitudes towards objects as predictors of single and multiple behavioral criteria. Psychol Rev.

[13] Steel RP, Ovalle NK (1984). A review and meta-analysis of research on the relationship between behavioral intentions and employee turnover. J Appl Psychol.

[14] Flinkman M, Leino-Kilpi H, Salanterä S (2010). Nurses’ intention to leave the profession: integrative review. J Adv Nurs.

[15] Program Overview - Midwifery Education Program - Ryerson University. [Cited 2019 Sep 4]. Available from: https://www.ryerson.ca/midwifery/program/.

[16] Midwifery | Program Details. [cited 2019 Sep 4]. Available from: https://laurentian.ca/program/midwifery/details.

[17] Duncan K (1997). Student pre-entry experience and first year of employment. J Contin Educ Nurs.

[18] Spouse J (2000). An impossible dream? Images of nursing held by pre-registration students and their effect on sustaining motivation to become nurses. J Adv Nurs.

[19] Bretz RD, Judge TA (1998). Realistic job previews: a test of the adverse self-selection hypothesis. J Appl Psychol.

[20] Hamshire C, Willgoss TG, Wibberley C (2012). “The placement was probably the tipping point”--the narratives of recently discontinued students. Nurse Educ Pract.

[21] Hamshire C, Willgoss TG, Wibberley C (2013). Should I stay or should I go? A study exploring why healthcare students consider leaving their programme. Nurse Educ Today.

[22] Urwin S, Stanley R, Jones M, Gallagher A, Wainwright P, Perkins A (2010). Understanding student nurse attrition: learning from the literature. Nurse Educ Today.

[23] McCall L, Wray N, McKenna L (2009). Influence of clinical placement on undergraduate midwifery students’ career intentions. Midwifery..

[24] Hayes LJ, Orchard CA, McGillis Hall L, Nincic V, O’Brien-Pallas L, Andrews G (2006). Career intentions of nursing students and new nurse graduates: a review of the literature. Int J Nurs Educ Scholarsh.

[25] Cameron J, Roxburgh M, Taylor J, Lauder W (2011). An integrative literature review of student retention in programmes of nursing and midwifery education: why do students stay?. J Clin Nurs.

[26] Eick SA, Williamson GR, Heath V (2012). A systematic review of placement-related attrition in nurse education. Int J Nurs Stud.

[27] Kahn RL, Wolfe DM, Quinn RP, Snoek JD. PsycNET. psycnet.apa.org; 1964 [cited 2018 Sep 20]. Available from: http://psycnet.apa.org/record/1965-08866-000.

[28] Fujimoto T, Kotani S, Suzuki R (2008). Work--family conflict of nurses in Japan. J Clin Nurs.

[29] Jamal M, Baba VV (1997). Shiftwork, burnout, and well-being: a study of Canadian nurses. Int J Stress Manag.

[30] Yildirim D, Aycan Z (2008). Nurses’ work demands and work--family conflict: a questionnaire survey. Int J Nurs Stud.

[31] Cinamon RG, Rich Y (2005). Work–family conflict among female teachers. Teach Teach Educ.

[32] Connell RW (2005). Work/life balance, gender equity and social change. Aust J Soc Issues.

[33] Gregory A, Milner S (2009). Work-life balance: a matter of choice?. Gend Work Organ.

[34] Goode WJ (1960). A theory of role strain. Am Sociol Rev.

[35] Karasek RA Jr. Job demands, job decision latitude, and mental strain: implications for job redesign. Adm Sci Q. 1979:285–308. 10.2307/2392498.

[36] Grandey AA, Cropanzano R (1999). The conservation of resources model applied to work–family conflict and strain. J Vocat Behav.

[37] Peluchette JVE (1993). Subjective career success: the influence of individual difference, family, and organizational variables. J Vocat Behav.

[38] Shuval JT, Gross SE (2008). Midwives practice CAM: feminism in the delivery room. Complement Health Pract Rev.

[39] Bloxsome D, Ireson D, Doleman G, Bayes S (2019). Factors associated with midwives’ job satisfaction and intention to stay in the profession: an integrative review. J Clin Nurs.

[40] Allen TD, Schmidt NW, Highhouse S, Irving IB (2013). The work-family role interface: a synthesis of the research from industrial and organizational psychology. Handbook of psychology.

[41] Marks SR. Multiple roles and role strain: some notes on human energy, time and commitment. Am Sociol Rev. 1977:921–36. 10.2307/2094577.

[42] Rothbard NP (2001). Enriching or depleting? The dynamics of engagement in work and family roles. Adm Sci Q.

[43] Edwards JR, Rothbard NP (2000). Mechanisms linking work and family: clarifying the relationship between work and family constructs. AMRO..

[44] Wayne JH, Grzywacz JG, Carlson DS, Kacmar KM (2007). Work–family facilitation: a theoretical explanation and model of primary antecedents and consequences. Hum Resour Manag Rev.

[45] Greenhaus JH, Powell GN (2006). When work and family are allies: a theory of work-family enrichment. AMRO..

[46] McNall LA, Nicklin JM, Masuda AD (2010). A meta-analytic review of the consequences associated with work–family enrichment. J Bus Psychol.

[47] Beck CT (1993). Nursing students’ initial clinical experience: a phenomenological study. Int J Nurs Stud.

[48] Kuokkanen L, Leino-Kilpi H, Katajisto J (2003). Nurse empowerment, job-related satisfaction, and organizational commitment. J Nurs Care Qual.

[49] Barron D, West E (2005). Leaving nursing: an event-history analysis of nurses’ careers. J Health Serv Res Policy.

[50] Laine M, Organizational and professional commitment of nurses (2005). Finish Institute of Occupational Health University of Turku, Faculty of Medicine and Occupational Health Finland.

[51] Lyons TF (1971). Role clarity, need for clarity, satisfaction, tension, and withdrawal. Org Behav Hum Perfor.

[52] Fisher GG. PsycNET. psycnet.apa.org; 2002 [cited 2018 Sep 20]. Available from: http://psycnet.apa.org/record/2002-95014-382.

[53] Leap N (2002). Identifying the midwifery practice component of Australian midwifery education programs. Results of the Australian midwifery action project (AMAP) education survey. Aust J Midwifery.

